# Incidence, prevalence, and management of MRSA bacteremia across patient populations—a review of recent developments in MRSA management and treatment

**DOI:** 10.1186/s13054-017-1801-3

**Published:** 2017-08-14

**Authors:** Ali Hassoun, Peter K. Linden, Bruce Friedman

**Affiliations:** 1 Alabama Infectious Disease Center, 420 Lowell Drive, Suite 301, Huntsville, AL 35801 USA; 2 Allegheny General Hospital, Division of Surgical Critical Care, Allegheny Professional Building, 490 East North Ave, Suite 309, Pittsburgh, PA 15212 USA; 3 Joseph M. Still Burn Center, 3675 J. Dewey Gray Circle, Suite 200B, Augusta, GA 30909 USA

**Keywords:** MRSA, MSSA, *Staphylococcus aureus*, Bacteremia, Epidemiology, Management, Incidence, Prevalence

## Abstract

Methicillin-resistant *Staphylococcus aureus* (MRSA) infection is still a major global healthcare problem. Of concern is *S. aureus* bacteremia, which exhibits high rates of morbidity and mortality and can cause metastatic or complicated infections such as infective endocarditis or sepsis. MRSA is responsible for most global *S. aureus* bacteremia cases, and compared with methicillin-sensitive *S. aureus*, MRSA infection is associated with poorer clinical outcomes. *S. aureus* virulence is affected by the unique combination of toxin and immune-modulatory gene products, which may differ by geographic location and healthcare- or community-associated acquisition. Management of *S. aureus* bacteremia involves timely identification of the infecting strain and source of infection, proper choice of antibiotic treatment, and robust prevention strategies. Resistance and nonsusceptibility to first-line antimicrobials combined with a lack of equally effective alternatives complicates MRSA bacteremia treatment. This review describes trends in epidemiology and factors that influence the incidence of MRSA bacteremia. Current and developing diagnostic tools, treatments, and prevention strategies are also discussed.

## Background

Antimicrobial resistance is a major global health concern, and, of the Gram-positive bacteria, drug-resistant *Staphylococcus aureus* is a serious threat [1, 2]. *S. aureus* causes a wide range of infections commonly involving the skin, soft tissue, bone, joints, and infections associated with indwelling catheters or prosthetic devices [3]. In addition, *S. aureus* is a leading cause of bacteremia in industrialized nations [4, 5]. Although methicillin-resistant *S. aureus* (MRSA) bacteremia incidence has decreased over the past decade [3], MRSA remains associated with poorer clinical outcomes compared with methicillin-sensitive *S. aureus* (MSSA) [6]. *S. aureus* bacteremia (SAB) often causes metastatic infections such as infective endocarditis (IE), septic arthritis, and osteomyelitis [3]. Moreover, SAB can lead to complications such as sepsis and septic shock [6]. Taken together, these issues make SAB particularly challenging to treat.

Choice and timing of antibacterial therapy greatly affect treatment outcomes in SAB [6]. For SAB caused by MSSA, β-lactam therapy is considered the gold standard [6, 7]. For MRSA, the 2011 Infectious Diseases Society of America guidelines recommend treatment with vancomycin or daptomycin [3, 8]. However, each antimicrobial agent has limitations. Several issues restrict the utility of vancomycin, including slow bactericidal activity, low tissue penetration, and increasing reports of resistance and failure [9–11]. While daptomycin is effective against MRSA bacteremia, treatment-emergent nonsusceptibility is concerning [12–14], and evidence suggests prior vancomycin treatment may encourage daptomycin resistance in *S. aureus* [15, 16]. Given the substantial morbidity and mortality associated with SAB [6] and the limitations of currently approved treatments, there is a need to identify alternative agents for the treatment of MRSA bacteremia. Time to effective treatment is largely dependent on pathogen identification [17]. Delays in diagnosing and treating SAB lead to poorer clinical outcomes [18]. Standard microbial identification techniques take between 48 and 72 h, while recently developed rapid diagnostic tests provide data within 3 h of collection [19]. By enabling optimized antimicrobial therapy, rapid diagnostic tests may lower mortality, hospitalization, and costs [20]. This review discusses the global incidence and prevalence, diagnostic methods, and current management strategies for SAB. We also briefly discuss another key part of MRSA infection management—prevention; however, an in depth discussion is beyond the scope of this review.

## Prevalence of MRSA bacteremia

The prevalence of MRSA infections, especially bacteremia, differs around the world. In 2014, the percentage of invasive MRSA isolates in Europe ranged from 0.9% in the Netherlands to 56% in Romania, with a population-weighted mean of 17.4% [21]. MRSA prevalence exhibits a north–south variation in Europe, with a higher proportion of resistant isolates in southern countries compared with northern countries [21]. Even though the proportion of MRSA isolates in Europe has decreased over time, 7 of the 29 European Union countries still report 25% or more of invasive *S. aureus* isolates as MRSA [21].

A review of 15 studies shows between 13 and 74% of worldwide *S. aureus* infections are MRSA [22]. The prevalence of *S. aureus* infections in countries of South and East Asia and the Western Pacific is difficult to ascertain; however, publications and national surveillance data from these regions identify *S. aureus* as a significant pathogen, with MRSA incidence ranging from 2.3 to 69.1% [1, 23]. In 2005, invasive MRSA infections in the US occurred at a rate of 31.8 per 100,000 people after adjustment for age, race, and gender, and 75% of these invasive MRSA infections involved SAB [24]. This is higher than the MRSA bacteremia rates reported in Canada from 2000 to 2004, which were 2.1, 1.6, and 3.6 per 100,000 people for Calgary, Victoria, and Sherbrooke, respectively [25]. Within a 1-year period (2011–2012), 12.3% of all healthcare-associated infections in Europe were caused by *S. aureus* [26]. In Cyprus, Italy, Portugal, and Romania, more than 60% of healthcare-associated *S. aureus* infections were identified as MRSA [26].

**Fig. 1:**
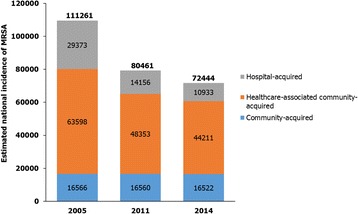
The national estimated number of MRSA infections in the US, stratified by infection setting. Adapted from data reported by the Center for Disease Control and Prevention [27] and Dantes et al. [28]. *MRSA* methicillin-resistant *S. aureus*

The origin of SAB cases—community-acquired, hospital-acquired, or healthcare-associated community onset—has been changing. The incidence of invasive MRSA infections in the US has decreased (Fig. 1) [27, 28], with healthcare-associated community-onset infections now making up the greatest proportion. Community-acquired MRSA bacteremia, including healthcare-associated community-onset, has superseded hospital-acquired MRSA bacteremia globally. Patients with healthcare-associated community-onset MRSA infections frequently have comorbidities, such as diabetes, decubitus, ulcers, chronic renal disease, prior stroke, or dementia [28]. Data from Canada, Australia, and Scandinavia show an increase in the rate of MRSA bacteremia between 2000 and 2008 (*P* = 0.035), mainly caused by an increase in community-acquired infections (*P* = 0.013). These findings indicate that community-acquired MRSA infections remain a threat.

## MRSA molecular epidemiology

Different strains are responsible for hospital- and community-acquired MRSA infections and can be identified using molecular typing methods such as pulsed field gel electrophoresis (PFGE) or multilocus sequence typing [29, 30]. These typing methods can distinguish strains based on the genes encoding the staphylococcus protein A or the staphylococcal chromosomal cassette (SCC) *mec* [29]. In the US, hospital-acquired MRSA infections are generally caused by the PFGE USA100 or USA200 strains, whereas community-acquired MRSA infections are commonly associated with the USA300 or USA400 strains [30]. Internationally, the ST239 strain is a common cause of hospital-acquired MRSA, but is rarely reported in the US [31, 32]. Methicillin resistance has been linked to clonal variants in the SCC*mec* gene [33]. Five different subtypes of SCC*mec* exist, which vary in size from about 20 to 68 kb [33]. Hospital-acquired MRSA strains often contain SCC*mec* subtype II [30, 34]. In contrast, SCC*mec* subtype IV, rarely found in hospital-acquired MRSA strains, is more prevalent in community-acquired MRSA strains globally [30, 34].

Other molecular features distinguish community-acquired from hospital-acquired strains of MRSA. Panton-Valentine leucocidin (PVL) is a powerful exotoxin that induces lysis of leukocytes, particularly neutrophils [35, 36]. Community-acquired strains, including the USA300 strain, usually carry the gene for PVL, whereas it is rare in hospital-acquired strains [30, 37]. The USA300 PVL-positive strain is not prevalent in Western Europe, where other PVL-positive strains, including ST80, are more commonly reported [38]. PVL-positive *S. aureus* strains have been responsible for outbreaks of necrotizing pneumonia and invasive skin disease; however, the role of PVL in disease severity is not clear [39]. PVL is not the only toxin produced by MRSA. Different strains produce a range of toxins, including toxic shock syndrome toxin-1 (TSST1), staphylococcal enterotoxin B or C, α-hemolysin, and the phenol-soluble modulins (PSMs) [40, 41]. The expression of several toxins, including α-hemolysin and PSMs, is controlled by the *agr* regulatory system, which is more likely to be present in community- than hospital-acquired strains [40]. Overall, the expression of toxin-producing genes is higher in community- than hospital-acquired MRSA strains and community-acquired strains tend to be more virulent [40, 42]. In patients with MRSA bacteremia, the presence of staphylococcal enterotoxin- and TSST1-producing genes is associated with a significant increase in the risk of mortality [41].

## MRSA colonization

About one-third of the general population is colonized with *S. aureus*, and the pooled prevalence of MRSA colonization is 1.3% (95% confidence interval [CI] 1.04–1.53%) [43]. MRSA colonization varied between studies depending on the methodology used. For example, when cultures were taken at the time of hospital admission or outpatient assessment, prevalence of colonization with community-acquired MRSA was 1.8%, but when samples were taken from individuals outside of the healthcare environment, it was 0.76% [43]. While the percentage of the US population with *S. aureus* nasal colonization has decreased over time, the proportion of people colonized with MRSA has increased [44]. Risk factors for colonization with MRSA in US females were age ≥60 years, diabetes, and poverty-level household income, whereas in US males the only significant risk factor was healthcare exposure [44]. Other studies identify chronic illness, injected drug use, recent hospitalization or outpatient visit, recent antibiotic use, and contact with an MRSA-infected person as risk factors.

While the most common site of MRSA colonization is the anterior nares [45], *S. aureus* (including MRSA) may also be present in the throat, axilla, rectum, groin, or perineum, and frequently colonizes more than one site [46, 47]. Recent studies suggest colonization of the throat is more prevalent than of the nose, and checking only the nose would fail to detect a significant portion of colonized persons [47, 48]. Regarding nasal carriage of *S. aureus*, about 20% of the population are persistently colonized with one strain, about 60% are intermittent carriers of varying strains, and the rest of the population never exhibit nasal colonization [49]. Hospital-acquired MRSA infections generally arise from persistent carriers undergoing antibiotic therapy or from intermittent carriers [45]. Both intermittent and persistent MRSA nasal colonization significantly increase the risk of developing an MRSA-invasive infection, including bacteremia (hazard ratios of 22.8 and 36.8, respectively; *P* value for both compared to noncarriers is <0.001) [50]. In a 2-year period, 21% of persistent and 13% of intermittent carriers developed an invasive infection [50].

To understand the relationship between colonizing and infecting strains, investigators analyzed patients admitted to the emergency department for closed skin abscesses [47]. The majority (~90%) were colonized with the *S. aureus* strain isolated from the infection, and 31% of these patients were colonized with an additional strain. Having two MRSA strains was uncommon (4.1%), but MRSA + MSSA or two MSSA strains were present in 20.4 and 22.2% of patients, respectively [47]. Concordance of the colonizing and infecting strain was also seen in about 82% of SAB patients [51].

## Detection and diagnosis of MRSA strains

Identifying the causative organism can be challenging in SAB, especially for resistant strains. Traditional culture and susceptibility testing for MRSA takes between 48 and 72 h, including a 16- to 24-h incubation and another 16 to 24 h to complete the susceptibility tests [19]. Recent advances in molecular and nonmolecular testing methods greatly reduced the time required to detect MRSA [19]. These rapid and sensitive screening assays could help to improve infection control and decrease costs. With a rapid test, Bauer et al. [20] observed bacteremia patients diagnosed with MRSA had a shorter length of stay and lower overall hospital costs, and for patients with MSSA, the switch from empiric to targeted therapy was 1.6 days shorter. Use of rapid molecular diagnostic tests rather than conventional methods is also associated with a significantly lower mortality risk for patients with bloodstream infections (odds ratio (OR) [95% CI] 0.66 [0.54–0.80]), including those caused by Gram-positive organisms (OR [95% CI] 0.73 [0.55–0.97]) [52]. Combining rapid molecular testing with an antibiotic stewardship program can further reduce the risk of mortality [52]. Individual hospitals deciding which test to implement must consider the specificity, sensitivity, price, turnaround time, and expertise required for each test [19, 53].

An adaptation to the traditional culture method is the use of chromogenic agar, which produces a color reaction in the bacterial cultures [19]. These media also contain antibiotics that only allow resistant bacteria to grow [19]. Thus, MRSA can be detected in 20 to 26 h [19]. A meta-analysis of performance characteristics of available chromogenic media tests reported a pooled sensitivity estimate of 78.3% after 18 to 24 h and of 87.6% after 48 h (Table 1), and the pooled specificity estimate was almost 97% at 18 to 24 h and 94% at 48 h [53]. Sensitivity at 18 to 24 h was significantly lower than at 48 h, but the sensitivity at 48 h was comparable between chromogenic media and traditional culture methods (86.9%) [53]. The 18- to 24-h specificity of chromogenic media for detecting MRSA was significantly higher than the specificity of traditional culture (Table 1) [53]. In clinical practice, the use of chromogenic media has been shown to reduce the time to targeted MRSA treatment by 12 h [17].

**Table 1:** Sensitivity and specificity of different MRSA testing methodologies based on pooled data

MRSA testing modality	Number of studies	Sensitivity, % (95% CI)	Specificity, % (95% CI)
Culture 48 h	7	86.9 (74.7–93.7)	89.7 (77.7–95.6)^a^
Chromogenic media, 18–24 h	28	78.3 (71.0–84.1)^a,b^	98.6 (97.7–99.1)^b,c^
Chromogenic media, 48 h	24	87.6 (82.1–91.6)	94.7 (91.6–96.8)
PCR	15	92.5 (87.4–95.9)	97.0 (94.5–98.4)

Adapted from data presented in Luteijn et al. [53]

*Abbreviations*: *CI* confidence interval, *MRSA* methicillin-resistant *S. aureus*, *PCR* polymerase chain reaction

^a^ *P* <0.05 vs PCR

^b^ *P* <0.05 vs chromogenic media at 48 h

^c^ *P* < 0.05 vs culture at 48 h

Another innovation in MRSA detection is the development of real-time polymerase chain reaction (PCR) tests capable of detecting genes specific to *S. aureus* [19]. To distinguish MRSA strains from MSSA or methicillin-resistant coagulase-negative staphylococci, PCR methods target a portion of DNA where the MRSA-specific SCC*mec* gene meets the *S. aureus orfX* gene [19]. The PCR tests can be performed directly on samples obtained from blood or a nasal or wound swab, and results are usually available within 1 to 3 h [19]. In clinical practice, however, turnaround times from sampling to a result are typically longer because of the time required to transport samples, conduct the test, and report the results [54]. Nevertheless, the overall time is generally much shorter with PCR-based assays than with chromogenic media culture [54]. In addition, PCR tests showed pooled estimates for sensitivity and specificity of 92.5 and 97.0%, respectively, in the meta-analysis mentioned above [53]. Furthermore, the sensitivity of PCR was significantly higher than that of chromogenic media, and the specificity was significantly higher than that of traditional culture [53]. Relative to MRSA detection by chromogenic agar, PCR reduced the overall duration of patient isolation and number of days patients were inappropriately isolated during their hospital stay [54].

Another approach to MRSA detection is to use immunochromatographic tests. These tests use antibodies toward specific bacterial proteins to generate a visible reaction in the test medium if that protein is present in the sample [19, 55]. One type of immunochromatographic test is the latex agglutination test, which uses a monoclonal antibody against PBP2a, a protein produced by the *mecA* gene [19]. If PBP2a is present in the sample, the latex particles sensitized by the antibody clump together, forming a readily visible agglutination [19]. The latex agglutination test has sensitivity of 97% for correctly identifying MRSA and a specificity of 100% for distinguishing MRSA from MSSA, even in low-level samples [19, 56]. Another PBP2a-antibody test (Clearview Exact) has identical performance characteristics in low-level MRSA samples (sensitivity 97% and specificity 100%) [56], but requires fewer steps than the latex agglutination test [57]. This test takes less than 6 minutes to complete, and results appear as colored lines on test strips [57]. The BinaxNOW *Staphylococcus aureus* Test differentiates *S. aureus* from coagulase-negative staphylococci and other Gram-positive cocci directly from positive blood culture bottles [55]. This test takes less than 30 minutes and has a sensitivity of 95.8% and a specificity of 99.6% [55]. While this test does not identify MRSA specifically, it can rule out other staphylococci and is inexpensive [55]. Because of the low cost and speed of results, it may be a useful test to undertake before sending samples for PCR testing [55].

## MRSA treatments and outcomes

Although MRSA bacteremia must always be taken seriously, some clinical characteristics place patients at risk of a complicated course requiring prolonged treatment (Table 2) [7, 58]. *S. aureus* bacteremia is considered uncomplicated when the infection meets the following requirements: a catheter-related infection where the catheter is removed; negative result on follow-up blood culture; fever resolution within 72 h; no abnormal findings on transesophageal echocardiogram; no implanted prosthetic material; and no symptoms of a metastatic infection [8, 58]. Complicated bacteremia is diagnosed when any of these criteria are not met. In general, antistaphylococcal treatment should be continued for about 14 days in those with uncomplicated bacteremia, and for 4 to 6 weeks in those with complicated SAB [8, 59].

**Table 2:** Demographic and clinical characteristics associated with more severe SAB

Characteristic	Impact
Community-acquired infection	Tends to metastasize
Female gender	Increased risk of mortality vs males
Positive blood cultures present for longer than 48 h	Complicated course (including metastatic infections)
Persistent fever at 72 h	Complicated course
Time for blood culture to turn positive	Complicated course (including metastatic infections and increased risk of mortality)
Lack of identifiable focus	Aggravates and prolongs SAB
Skin lesions suggestive of acute systemic infection	Complicated course
Implanted prosthetic device	Complicated course (including increased risk of mortality and relapse)
Immunosuppression and HIV	Aggravates and prolongs SAB
Renal failure	Intravascular complications
Solid tumors	Intravascular complications
APACHE II score >7	Complicated course (including increased risk of septic shock and mortality)
CURB-65 score >3	Complicated course (including increased risk of septic shock and mortality)
Neurologic complications	Increased risk of mortality
Cardiac complications	Increased risk of mortality
Septic thrombophlebitis	Prolonged clinical course
MRSA pneumonia	Complicated course (including increased risk of septic shock and mortality)

Portions of this table were reproduced with permission from Table 1 in Keynan and Rubinstein [7] and additional information was adapted from Corey [58]

Abbreviations: *APACHE* Acute Physiology and Chronic Health Evaluation, *CURB-65* confusion, urea, respiratory rate, blood pressure, and age 65, *HIV* human immunodeficiency virus, *MRSA* methicillin-resistant *S. aureus*, *SAB S. aureus* bacteremia

An important first step in MRSA bacteremia management is to identify and eliminate the source of infection [8, 60]. If an intravascular catheter is the source, it should be removed as soon as possible after diagnosis; any wounds should be debrided [8, 60]. In patients with short- or long-term catheter-related MRSA infection who develop suppurative thrombophlebitis, remove the catheter and treat as complicated bacteremia; anticoagulation with heparin has been reported, but evidence to support a recommendation is lacking [61]. Patients with MRSA IE and a prosthetic valve should be evaluated for valve replacement surgery, as should those with native valve IE if the infection is extensive or complicated [8]. When bacteremia is persistent, hidden sources of infection should be identified using MRI or CT imaging and removed by drainage or surgical debridement [8].

Empirical treatment decisions in MRSA bacteremia require consideration of the prevalence and resistance profile of local strains, risk factors for a complicated clinical course, presence of comorbidities, concurrent interventions, and response to prior antibiotics [59]. Current US and European treatment recommendations are summarized in Table 3 [8, 59, 60, 62, 63]. For most cases of MRSA bacteremia, vancomycin or daptomycin is the recommended treatment [64]. The choice of antibiotic may also depend on if the bacteremia is secondary to another infection. For example, daptomycin, although indicated for treatment of SAB, is contraindicated for SAB originating from pneumonia since pulmonary surfactants inactivate it [3]. Vancomycin poorly penetrates lung tissue; thus, linezolid or clindamycin are recommended if the strain is susceptible [8]. Reports of MRSA isolates resistant or nonsusceptible to currently available antibiotics, including vancomycin [11, 59], daptomycin [65], and ceftaroline [66], as well as multidrug-resistant MRSA clones, are a concerning trend [67]. These data highlight the importance of early identification of MRSA and susceptibility to identify the optimal antibiotic.

**Table 3:** Treatment recommendations for MRSA bacteremia

Condition	IDSA [8]	ESCMID/ISC/ESC [59, 60, 62, 63]
Uncomplicated bacteremia	Vancomycin or daptomycin 6 mg/kg/dose IV once daily for 2 weeks	Vancomycin doses to trough plasma concentration of 15–20 mg/L or teicoplanin if nephrotoxicity is a concern (daptomycin if vancomycin is poorly tolerated) for 10–14 days Consider switching to linezolid PO in patients with a rapid response and negative cultures after catheter removal
Complicated bacteremia	Vancomycin or daptomycin 6 mg/kg/dose IV once daily for 4–6 weeks, depending on extent of infection	Vancomycin, but switch to daptomycin if there is poor response or use daptomycin first-line in patients with life-threatening infection, renal impairment, previous glycopeptide use, or vancomycin resistance or reduced susceptibility Treat for 4–6 weeks
Infective endocarditis, native valve	Vancomycin or daptomycin 6 mg/kg/dose IV once daily for 6 weeks	Vancomycin 30–60 mg/kg/day IV in 2–3 doses for 4–6 weeks Alternative therapies: daptomycin 10 mg/kg/day IV once daily for 4–6 weeks or TMP/SMX + clindamycin
Infective endocarditis, prosthetic valve	Vancomycin IV + rifampin 300 mg PO/IV for ≥6 weeks + gentamicin 1 mg/kg/dose IV q8h for 2 weeks	Vancomycin 30–60 mg/kg/day IV in 2–3 doses for ≥6 weeks + rifampin 900–1200 mg IV or orally in 2–3 doses for ≥6 weeks and gentamicin 3/mg/kg/day IV or IM in 1–2 doses for 2 weeks
Infective endocarditis, right-sided		Vancomycin 15 mg/kg q12h for 6 weeks or daptomycin ≥6 mg/kg/day for 4–6 weeks if patient has renal impairment, sustained bacteremia for >7 days, infection with a VISA strain Optional addition of short-term gentamicin to vancomycin Alternative option: vancomycin + rifampin
Infective endocarditis, left-sided		Vancomycin 15 mg/kg q12h for 4–6 weeks with early and careful attention to culture results Switch to high-dose daptomycin (10 mg/kg/day) if no response to vancomycin and isolate is susceptible Optional addition of short-term gentamicin to vancomycin Alternative option: vancomycin + rifampin
Persistent bacteremia, despite vancomycin treatment	If isolate is susceptible, high-dose daptomycin (10 mg/kg/day) + another agent^a^ If isolate has reduced susceptibility to vancomycin and daptomycin, options for monotherapy or combination therapy are quinupristin/dalfopristin 7.5 mg/kg/dose IV q8h, linezolid 600 mg PO/IV bid, or telavancin 10 mg/kg/dose IV od	Daptomycin 10 mg/kg/day if isolates susceptible, possibly in combination with another agent (e.g., gentamicin, rifampicin, linezolid, a beta-lactam, or trimethoprim-sulfamethoxazole) Options for agents with reduced susceptibility to daptomycin or vancomycin, including quinupristin/dalfopristin, linezolid, or telavancin

Adapted from US and International guidelines and recommendations found in Garau et al. [60], Gould et al. 2011 [59], Gould et al. 2012 [62], Habib et al. [63], and Liu et al. [8]

^a^Options include gentamicin 1 mg/kg IV q8h, rifampin 600 mg PO/IV daily or 300–450 mg PO/IV bid, linezolid 600 mg PO/IV bid, trimethoprim-sulfamethoxazole 5 mg/kg IV bid, or a beta-lactam antibiotic

*Abbreviations*: *bid* twice daily, *ESC* European Society of Cardiology, *ESCMID* European Society of Clinical Microbiology and Infectious Diseases, *IDSA* Infectious Disease Society of America, *ISC* International Society of Chemotherapy, *IM* intramuscular, *IV* intravenous, *MRSA* methicillin-resistant *S. aureus*, *od* once daily, *PO* orally, *q8h*/*q12h* every 8/12 h, *TMP*/*SMX* trimethoprim/sulfamethoxazole, *VISA* vancomycin-intermediate *S. aureus*

Although vancomycin is the first-line antibiotic for MRSA bacteremia treatment, it has a relatively slow onset of bactericidal activity and poorly penetrates some tissues [68]. While US guidelines recommend a fixed dose, European guidelines advise dosing vancomycin based on the trough plasma concentration (C_min_) [62] with the goal to achieve a vancomycin area under the curve to minimum inhibitory concentration (MIC) ratio ≥400 for as long as possible throughout the 24-h dosing interval [10]. However, recent evidence suggests C_min_ is not an accurate surrogate for 24-h vancomycin exposure, underestimating the area under the curve by up to 25% [65]. In an analysis of MRSA bacteremia cases that received vancomycin, those who achieved a C_min_ of 15 to 20 mg/L within 72 h had a significantly lower rate of vancomycin failure compared with lower C_min_ values, but 40% of patients who had a C_min_ in the recommended range still did not [68]. This may reflect the observed slow increase in the MIC (MIC creep) of vancomycin from the 1990s to the present, whereby higher doses are needed to maintain efficacy [15]. Reports conflict on the correlation between vancomycin MICs >1.5 mg/L and treatment failure in MRSA bacteremia [69, 70]. Additionally, individual studies may be affected by the method used to determine MIC (Etest or broth microdilution) or by duration of storage of isolates [71]. Another concern of using higher doses of vancomycin is the potential for nephrotoxicity [68], a risk factor for mortality in SAB [72]. In a recent meta-analysis, continuous infusion of vancomycin was associated with less risk of nephrotoxicity compared to intermittent infusion, but no significant difference was found for mortality [73]. However, Echeverria-Esnal et al. [74] highlight factors that affect vancomycin-induced nephrotoxicity not considered in the individual studies, and suggest a multicenter randomized trial is needed to resolve the inconsistencies.

Daptomycin is considered an alternative first-line agent for MRSA bacteremia [64], but MICs for vancomycin and daptomycin are correlated [59, 65], and up to 15% of heterogenous vancomycin-intermediate *S. aureus* isolates are also nonsusceptible to daptomycin [65]. Furthermore, some studies suggest prior vancomycin failure is correlated with the acquisition of heteroresistance and reduced success of daptomycin therapy [12, 15, 16]. Thus, higher doses of daptomycin (8–10 mg/kg) may be required for complicated or persistent MRSA bacteremia [65]. Teicoplanin is another option for patients who are refractory to vancomycin; however, it is unavailable in some markets, including the US [64]. It is approved by the European Medicines Agency for use in bacteremia associated with several Gram-positive infections, and is considered as effective and safe as vancomycin in treating healthcare-associated MRSA bacteremia [75].

Given the limitations of currently approved treatments, other options are being developed. Vaccines targeting one or more *S. aureus* antigens have had minimal success to date and are reviewed elsewhere [76]. Several studies have evaluated alternative antibacterials, including ceftaroline, linezolid, and quinupristin/dalfopristin (Q/D), although none have been approved for treatment of MRSA bacteremia [64]. Ceftaroline is indicated for treatment of acute bacterial skin and skin structure infections and community-acquired bacterial pneumonia caused by *S. aureus*, but is often used off-label to treat SAB. A recent multicenter study found that approximately 70% of patients with MRSA bacteremia experienced clinical success when ceftaroline was used as a salvage therapy alone or in combination with another antistaphylococcal antibiotic [77]. Clinical trials of ceftaroline compared to other MRSA bacteremia antimicrobials are still needed. Linezolid, indicated for pneumonia and complicated and uncomplicated skin and skin structure infections caused by *S. aureus*, was effective as a salvage therapy for MRSA bacteremia [78, 79]. It is bacteriostatic against staphylococci, while the other treatments are bactericidal. Quinupristin/dalfopristin is indicated for treatment of complicated skin and skin structure infections (cSSSI) caused by MSSA, but is known to have in vitro activity against MRSA. In a study using Q/D as salvage therapy for 12 patients with MRSA or methicillin-resistant *S. epidermis* infections that did not respond to vancomycin, five of seven MRSA bacteremic patients showed eradication of the bacteria [80]. Telavancin is approved for use in Gram-positive cSSSI and hospital-acquired and ventilator-associated bacterial pneumonia (HABP/VABP), and it is currently being evaluated for treatment of *S. aureus* bacteremia in a phase 3 trial (NCT02208063). In the clinical trials comparing telavancin to vancomycin, clinical cure rates for patients with cSSSI or HABP/VABP with baseline MRSA bacteremia were 61.5 and 52.4% for telavancin-treated patients and 50.0 and 37.5% in vancomycin-treated patients, respectively [81]. Trimethoprim/sulfamethoxazole has also been suggested as an alternative treatment; however, it failed to meet noninferiority criteria compared with vancomycin in several trials of severe MRSA infections, including SAB [64, 82].

Combination therapy is another option being explored. Davis et al. [83] compared vancomycin plus flucloxacillin to vancomycin alone in 60 MRSA bacteremia patients. Duration of bacteremia was reduced by 1 day and fewer combination therapy patients had persistent bacteremia at 3 and 7 days. Combination of daptomycin and ceftaroline retained a bactericidal effect on isolates that had increased daptomycin MICs [84]. A phase 3 trial investigating β-lactam antibiotics given with daptomycin or vancomycin for MRSA bacteremia is ongoing (NCT02365493). Certain patients with IE may also benefit from combination of vancomycin or daptomycin with rifampin or an aminoglycoside. Although there are no definitive studies supporting its use, the addition of short-term gentamicin or rifampin is recommended in patients with prosthetic valve or left-sided disease [8, 60]; however, the European Society of Cardiology recommends against the use of an aminoglycoside in *S. aureus* native valve IE due to increased renal toxicity [63]. Rifampin is bactericidal and can penetrate biofilms; however, it should not be used alone due to high potential to induce resistance [8]. Any benefits of combination therapies should be carefully weighed against the probable effects on the intestinal microbiota, development of multidrug-resistant microorganisms, and possibly defying the protocols established by antimicrobial stewardship programs.

## Transmission prevention strategies

All healthcare personnel interacting with an MRSA-infected or -colonized person should use contact precautions to limit spread between patients [85]. This means putting the MRSA-infected patient into a single or private room, and wearing gowns and gloves when entering the patient’s room and removing them before exiting [85]. Since MRSA colonization can be persistent, contact precautions should be used throughout an infected person’s hospitalization (even after they have recovered from the MRSA infection) and with any person with a history of MRSA infection [85]. Ideally, healthcare facilities should have a system in place to alert them to the readmission or transfer of an MRSA-infected patient, so appropriate controls can be put in place on their arrival [85]. Hospital-wide hand hygiene campaigns have also greatly contributed to reduction of MRSA infections (reviewed in [86]).

Because MRSA can contaminate the environment, the rooms of MRSA-infected patients require strict disinfection of furniture, overbed tables, handrails, sinks, floors, and any healthcare equipment used during patient care (e.g., stethoscopes, thermometers, blood pressure cuffs) [85]. Xenon-UV light alone or in combination with normal cleaning decreases the presence of MRSA and other pathogens on surfaces by up to 99% [87]. Use of certain materials such as copper alloys in building design can also reduce the environmental burden and transmission of MRSA and other hospital-acquired pathogens [88].

Hospitals with high rates of MRSA infection should implement an active surveillance program to identify asymptomatic MRSA carriers and targeted MRSA decolonization programs to reduce infection rates [85]. Surveillance combined with prophylactic treatment has been very effective in reducing surgical site infections [89]. These protocols may combine intranasal antibiotics such as mupirocin with an antiseptic body wash or preoperative antibiotics [89]. Surveillance is the key, though, to prevent misuse and overuse of antibiotics [89].

## Conclusions

Although identification and prevention techniques have improved, MRSA remains a major healthcare issue. MRSA bacteremia can be challenging to manage, especially in patients at high risk of complications or in those with toxigenic or multidrug-resistant strains. Early identification of MRSA is an important step toward timely implementation of appropriate treatment. The development of new molecular and immunochromatographic testing technologies has the potential to dramatically shorten delays to diagnosis and treatment. In addition, novel antibiotic therapies are becoming available to provide effective alternatives for strains that have acquired resistance to existing drugs. While these advances do not preclude the need for vigilance and effective MRSA prevention strategies, they help mitigate some of the challenges associated with MRSA bacteremia treatment.

## Abbreviations

CT: Computed tomography
HABP/VABP: Hospital-acquired and ventilator-associated bacterial pneumonia
IE: Infective endocarditis
MIC: Minimum inhibitory concentration
MRI: Magnetic resonance imaging
MRSA: Methicillin-resistant *Staphylococcus aureus*
MSSA: Methicillin-sensitive *Staphylococcus aureus*
PCR: Polymerase chain reaction
PFGE: Pulsed field gel electrophoresis
PSM: Phenol-soluble modulin
PVL: Panton-Valentine leukocidin
Q/D: Quinupristin/dalfopristin
SAB: *Staphylococcus aureus* bacteremia
SCC: Staphylococcal chromosomal cassette
TMP/SMX: Trimethoprim/sulfamethoxazole
TSST1: Toxic shock syndrome toxin-1
VISA: Vancomycin-intermediate *Staphylococcus aureus*

## References

[1] World Health Organization. WHO Antimicrobial Resistance: Global Report on Surveillance; 2014. http://www.who.int/drugresistance/documents/surveillancereport/en/. Accessed 28 Oct 2016.

[2] Centers for Disease Control and Prevention. Antibiotic resistance threats in the United States; 2013. https://www.cdc.gov/drugresistance/threat-report-2013/. Accessed 17 Mar 2016.

[3] Tong SY, Davis JS, Eichenberger E, Holland TL, Fowler Jr VG. *Staphylococcus aureus* infections: epidemiology, pathophysiology, clinical manifestations, and management. Clin Microbiol Rev. 2015;28:603–61. 10.1128/CMR.00134-14 PMC4451395 26016486

[4] Weiner LM, Webb AK, Limbago B, Dudeck MA, Patel J, Kallen AJ (2016). Antimicrobial-resistant pathogens associated with healthcare-associated infections: summary of data reported to the National Healthcare Safety Network at the Centers for Disease Control and Prevention, 2011–2014. Infect Control Hosp Epidemiol..

[5] Laupland KB (2013). Incidence of bloodstream infection: a review of population-based studies. Clin Microbiol Infect..

[6] van Hal SJ, Jensen SO, Vaska VL, Espedido BA, Paterson DL, Gosbell IB. Predictors of mortality in *Staphylococcus aureus* Bacteremia. Clin Microbiol Rev. 2012;25:362–86. 10.1128/CMR.05022-11 PMC3346297 22491776

[7] Keynan Y, Rubinstein E. *Staphylococcus aureus* bacteremia, risk factors, complications, and management. Crit Care Clin. 2013;29:547–62. 10.1016/j.ccc.2013.03.008 23830653

[8] Liu C, Bayer A, Cosgrove SE, Daum RS, Fridkin SK, Gorwitz RJ, et al. Clinical practice guidelines by the Infectious Diseases Society of America for the treatment of methicillin-resistant *Staphylococcus aureus* infections in adults and children: executive summary. Clin Infect Dis. 2011;52:285–92. 10.1093/cid/cir034 21217178

[9] Lamp KC, Rybak MJ, Bailey EM, Kaatz GW (1992). In vitro pharmacodynamic effects of concentration, pH, and growth phase on serum bactericidal activities of daptomycin and vancomycin. Antimicrob Agents Chemother..

[10] Rybak M, Lomaestro B, Rotschafer JC, Moellering R, Craig W, Billeter M (2009). Therapeutic monitoring of vancomycin in adult patients: a consensus review of the American Society of Health-System Pharmacists, the Infectious Diseases Society of America, and the Society of Infectious Diseases Pharmacists. Am J Health Syst Pharm..

[11] Han JH, Edelstein PH, Lautenbach E. Reduced vancomycin susceptibility and staphylococcal cassette chromosome *mec* (SCC*mec*) type distribution in methicillin-resistant *Staphylococcus aureus* bacteraemia. J Antimicrob Chemother. 2012;67:2346–9. 10.1093/jac/dks255 PMC3444231 22761330

[12] Kullar R, Casapao AM, Davis SL, Levine DP, Zhao JJ, Crank CW (2013). A multicentre evaluation of the effectiveness and safety of high-dose daptomycin for the treatment of infective endocarditis. J Antimicrob Chemother..

[13] Sharma M, Riederer K, Chase P, Khatib R. High rate of decreasing daptomycin susceptibility during the treatment of persistent *Staphylococcus aureus* bacteremia. Eur J Clin Microbiol Infect Dis. 2008;27:433–7. 10.1007/s10096-007-0455-5 18214559

[14] Moore CL, Osaki-Kiyan P, Haque NZ, Perri MB, Donabedian S, Zervos MJ. Daptomycin versus vancomycin for bloodstream infections due to methicillin-resistant *Staphylococcus aureus* with a high vancomycin minimum inhibitory concentration: a case-control study. Clin Infect Dis. 2012;54:51–8. 10.1093/cid/cir764 22109947

[15] Moise PA, Amodio-Groton M, Rashid M, Lamp KC, Hoffman-Roberts HL, Sakoulas G, et al. Multicenter evaluation of the clinical outcomes of daptomycin with and without concomitant beta-lactams in patients with *Staphylococcus aureus* bacteremia and mild to moderate renal impairment. Antimicrob Agents Chemother. 2013;57:1192–200. 10.1128/AAC.02192-12 PMC3591880 23254428

[16] Sakoulas G, Alder J, Thauvin-Eliopoulos C, Moellering Jr RC, Eliopoulos GM. Induction of daptomycin heterogeneous susceptibility in *Staphylococcus aureus* by exposure to vancomycin. Antimicrob Agents Chemother. 2006;50:1581–5. 10.1128/AAC.50.4.1581-1585.2006 PMC1426932 16569891

[17] Nicolsen NC, LeCroy N, Alby K, Martin KE, Laux J, Lin FC, et al. Clinical outcomes with rapid detection of methicillin-resistant and methicillin-susceptible *Staphylococcus aureus* isolates from routine blood cultures. J Clin Microbiol. 2013;51:4126–9. 10.1128/JCM.01667-13 PMC3838081 24088861

[18] Lodise TP, McKinnon PS, Swiderski L, Rybak MJ. Outcomes analysis of delayed antibiotic treatment for hospital-acquired *Staphylococcus aureus* bacteremia. Clin Infect Dis. 2003;36:1418–23. 10.1086/375057 12766837

[19] Palavecino EL (2014). Rapid methods for detection of MRSA in clinical specimens. Methods Mol Biol..

[20] Bauer KA, West JE, Balada-Llasat J-M, Pancholi P, Stevenson KB, Goff DA (2010). An antimicrobial stewardship program's impact. Clin Infect Dis..

[21] European Centre for Disease Prevention and Control. Antimicrobial resistance surveillance in Europe in 2014. Annual report of the European Antimicrobial Resistance Surveillance Network (EARS-Net). 2015. http://ecdc.europa.eu/en/publications/Publications/antimicrobial-resistance-europe-2014.pdf. Accessed 17 Aug 2016.

[22] Kock R, Becker K, Cookson B, van Gemert-Pijnen JE, Harbarth S, Kluytmans J, et al. Methicillin-resistant *Staphylococcus aureus* (MRSA): burden of disease and control challenges in Europe. Euro Surveill. 2010;15:19688. 10.2807/ese.15.41.19688-en 20961515

[23] Nickerson EK, West TE, Day NP, Peacock SJ. *Staphylococcus aureus* disease and drug resistance in resource-limited countries in south and east Asia. Lancet Infect Dis. 2009;9:130–5. 10.1016/S1473-3099(09)70022-2 19179228

[24] Klevens RM, Morrison MA, Nadle J, Petit S, Gershman K, Ray S. Invasive methicillin-resistant *Staphylococcus aureus* infections in the United States. JAMA. 2007;298:1763–71. 10.1001/jama.298.15.1763 17940231

[25] Laupland KB, Lyytikainen O, Sogaard M, Kennedy KJ, Knudsen JD, Ostergaard C, et al. The changing epidemiology of *Staphylococcus aureus* bloodstream infection: a multinational population-based surveillance study. Clin Microbiol Infect. 2013;19:465–71. 10.1111/j.1469-0691.2012.03903.x 22616816

[26] European Centre for Disease Prevention and Control. Point prevalence survey of healthcare-associated infections and antimicrobial use in European acute care hospitals, 2011-2012. 2013. http://ecdc.europa.eu/en/publications/Publications/healthcare-associated-infections-antimicrobial-use-PPS.pdf. Accessed 17 Aug 2016. 10.2807/ese.17.46.20316-en 23171822

[27] Centers for Disease Control and Prevention. Active bacterial core surveillance report, emerging infections program network, methicillin-resistant *Staphylococcus aureus*, 2014. https://www.cdc.gov/abcs/reports-findings/survreports/mrsa14.pdf. Accessed 15 Aug 2014.

[28] Dantes R, Mu Y, Belflower R, Aragon D, Dumyati G, Harrison LH, et al. National burden of invasive methicillin-resistant *Staphylococcus aureus* infections, United States, 2011. JAMA Intern Med. 2013;173:1970–8. 10.1001/jamainternmed.2013.10423 PMC10887428 24043270

[29] Tenover FC, McDougal LK, Goering RV, Killgore G, Projan SJ, Patel JB, et al. Characterization of a strain of community-associated methicillin-resistant *Staphylococcus aureus* widely disseminated in the United States. J Clin Microbiol. 2006;44:108–18. 10.1128/JCM.44.1.108-118.2006 PMC1351972 16390957

[30] Weber JT. Community-associated methicillin-resistant *Staphylococcus aureus*. Clin Infect Dis. 2005;41 Suppl 4:S269–72. 10.1086/430788 16032563

[31] Wang SH, Khan Y, Hines L, Mediavilla JR, Zhang L, Chen L, et al. Methicillin-resistant *Staphylococcus aureus* sequence type 239-III, Ohio, USA, 2007-2009. Emerg Infect Dis. 2012;18:1557–65. 10.3201/eid1810.120468 PMC3471631 23018025

[32] Xiao M, Wang H, Zhao Y, Mao LL, Brown M, Yu YS, et al. National surveillance of methicillin-resistant *Staphylococcus aureus* in China highlights a still-evolving epidemiology with 15 novel emerging multilocus sequence types. J Clin Microbiol. 2013;51:3638–44. 10.1128/JCM.01375-13 PMC3889745 23985906

[33] Appelbaum PC. Microbiology of antibiotic resistance in *Staphylococcus aureus*. Clin Infect Dis. 2007;45 Suppl 3:S165–70. 10.1086/519474 17712742

[34] Naimi TS, LeDell KH, Como-Sabetti K, Borchardt SM, Boxrud DJ, Etienne J, et al. Comparison of community- and health care-associated methicillin-resistant *Staphylococcus aureus* infection. JAMA. 2003;290:2976–84. 10.1001/jama.290.22.2976 14665659

[35] Meyer F, Girardot R, Piemont Y, Prevost G, Colin DA (2009). Analysis of the specificity of Panton-Valentine leucocidin and gamma-hemolysin F component binding. Infect Immun..

[36] Gauduchon V, Werner S, Prevost G, Monteil H, Colin DA (2001). Flow cytometric determination of Panton-Valentine leucocidin S component binding. Infect Immun..

[37] Vandenesch F, Naimi T, Enright MC, Lina G, Nimmo GR, Heffernan H, et al. Community-acquired methicillin-resistant *Staphylococcus aureus *carrying Panton-Valentine leukocidin genes: worldwide emergence. Emerg Infect Dis. 2003;9:978–84. 10.3201/eid0908.030089 PMC3020611 12967497

[38] Stefani S, Chung DR, Lindsay JA, Friedrich AW, Kearns AM, Westh H, et al. Meticillin-resistant *Staphylococcus aureus* (MRSA): global epidemiology and harmonisation of typing methods. Int J Antimicrob Agents. 2012;39:273–82. 10.1016/j.ijantimicag.2011.09.030 22230333

[39] Gordon RJ, Lowy FD. Pathogenesis of methicillin-resistant *Staphylococcus aureus* infection. Clin Infect Dis. 2008;46 Suppl 5:S350–9. 10.1086/533591 PMC2474459 18462090

[40] Diep BA, Otto M (2008). The role of virulence determinants in community-associated MRSA pathogenesis. Trends Microbiol.

[41] Maeda M, Shoji H, Shirakura T, Takuma T, Ugajin K, Fukuchi K, et al. Analysis of staphylococcal toxins and clinical outcomes of methicillin-resistant *Staphylococcus aureus* bacteremia. Biol Pharm Bull. 2016;39:1195–200. 10.1248/bpb.b16-00255 27374293

[42] Cosgrove SE, Sakoulas G, Perencevich EN, Schwaber MJ, Karchmer AW, Carmeli Y. Comparison of mortality associated with methicillin-resistant and methicillin-susceptible *Staphylococcus aureus* bacteremia: a meta-analysis. Clin Infect Dis. 2003;36:53–9. 10.1086/345476 12491202

[43] Salgado CD, Farr BM, Calfee DP. Community-acquired methicillin-resistant *Staphylococcus aureus*: a meta-analysis of prevalence and risk factors. Clin Infect Dis. 2003;36:131–9. 10.1086/345436 12522744

[44] Gorwitz RJ, Kruszon-Moran D, McAllister SK, McQuillan G, McDougal LK, Fosheim GE, et al. Changes in the prevalence of nasal colonization with *Staphylococcus aureus* in the United States, 2001-2004. J Infect Dis. 2008;197:1226–34. 10.1086/533494 18422434

[45] Kluytmans J, van Belkum A, Verbrugh H. Nasal carriage of *Staphylococcus aureus*: epidemiology, underlying mechanisms, and associated risks. Clin Microbiol Rev. 1997;10:505–20. 10.1128/cmr.10.3.505 PMC172932 9227864

[46] Mermel LA, Cartony JM, Covington P, Maxey G, Morse D. Methicillin-resistant *Staphylococcus aureus* colonization at different body sites: a prospective, quantitative analysis. J Clin Microbiol. 2011;49:1119–21. 10.1128/JCM.02601-10 PMC3067701 21209169

[47] Albrecht VS, Limbago BM, Moran GJ, Krishnadasan A, Gorwitz RJ, McDougal LK, et al. *Staphylococcus aureus* colonization and strain type at various body sites among patients with a closed abscess and uninfected controls at U.S. emergency departments. J Clin Microbiol. 2015;53:3478–84. 10.1128/JCM.01371-15 PMC4609677 26292314

[48] Kumar N, David MZ, Boyle-Vavra S, Sieth J, Daum RS. High *Staphylococcus aureus *colonization prevalence among patients with skin and soft tissue infections and controls in an urban emergency department. J Clin Microbiol. 2015;53:810–5. 10.1128/JCM.03221-14 PMC4390638 25540401

[49] Williams RE. Healthy carriage of *Staphylococcus aureus*: its prevalence and importance. Bacteriol Rev. 1963;27:56–71. 10.1128/br.27.1.56-71.1963 PMC441169 14000926

[50] Vigil DI, Harden WD, Hines AE, Hosokawa PW, Henderson WG, Bessesen MT (2015). Risk of MRSA infection in patients with intermittent versus persistent MRSA nares colonization. Infect Control Hosp Epidemiol.

[51] von Eiff C, Becker K, Machka K, Stammer H, Peters G. Nasal carriage as a source of *Staphylococcus aureus* bacteremia. Study Group. N Engl J Med. 2001;344:11–6. 10.1056/NEJM200101043440102 11136954

[52] Timbrook TT, Morton JB, McConeghy KW, Caffrey AR, Mylonakis E, LaPlante KL (2017). The effect of molecular rapid diagnostic testing on clinical outcomes in bloodstream infections: a systematic review and meta-analysis. Clin Infect Dis.

[53] Luteijn JM, Hubben GA, Pechlivanoglou P, Bonten MJ, Postma MJ. Diagnostic accuracy of culture-based and PCR-based detection tests for methicillin-resistant *Staphylococcus aureus*: a meta-analysis. Clin Microbiol Infect. 2011;17:146–54. 10.1111/j.1469-0691.2010.03202.x 20219085

[54] Polisena J, Chen S, Cimon K, McGill S, Forward K, Gardam M. Clinical effectiveness of rapid tests for methicillin resistant *Staphylococcus aureus * (MRSA) in hospitalized patients: a systematic review. BMC Infect Dis. 2011;11:336. 10.1186/1471-2334-11-336 PMC3259066 22151575

[55] Dhiman N, Trienski TL, DiPersio LP, DiPersio JR. Evaluation of the BinaxNOW *Staphylococcus aureus* test for rapid identification of Gram-positive cocci from VersaTREK blood culture bottles. J Clin Microbiol. 2013;51:2939–42. 10.1128/JCM.01087-13 PMC3754664 23804393

[56] Nonhoff C, Roisin S, Hallin M, Denis O. Evaluation of Clearview Exact PBP2a, a new immunochromatographic assay, for detection of low-level methicillin-resistant *Staphylococcus aureus* (LL-MRSA). J Clin Microbiol. 2012;50:3359–60. 10.1128/JCM.01829-12 PMC3457447 22814472

[57] van Meensel B, Frans J, Laffut W, Van Kerkhoven D, Lemmens A, Van Schaeren J, et al. Multicenter validation of the Clearview Exact PBP2a test. In: 21st European Congress of Clinical Microbiology and Infectious Diseases. Milan, Italy; 2011.

[58] Corey GR. *Staphylococcus aureus* bloodstream infections: definitions and treatment. Clin Infect Dis. 2009;48:S254–S9. 10.1086/598186 19374581

[59] Gould IM, Cauda R, Esposito S, Gudiol F, Mazzei T, Garau J. Management of serious meticillin-resistant *Staphylococcus aureus* infections: what are the limits? Int J Antimicrob Agents. 2011;37:202–9. 10.1016/j.ijantimicag.2010.10.030 21300528

[60] Garau J, Bouza E, Chastre J, Gudiol F, Harbarth S. Management of methicillin-resistant *Staphylococcus aureus* infections. Clin Microbiol Infect. 2009;15:125–36. 10.1111/j.1469-0691.2009.02701.x 19291144

[61] Mermel LA, Allon M, Bouza E, Craven DE, Flynn P, O'Grady NP (2009). Clinical practice guidelines for the diagnosis and management of intravascular catheter-related infection: 2009 Update by the Infectious Diseases Society of America. Clin Infect Dis.

[62] Gould IM, David MZ, Esposito S, Garau J, Lina G, Mazzei T, et al. New insights into meticillin-resistant *Staphylococcus aureus* (MRSA) pathogenesis, treatment and resistance. Int J Antimicrob Agents. 2012;39:96–104. 10.1016/j.ijantimicag.2011.09.028 22196394

[63] Habib G, Lancellotti P, Antunes MJ, Bongiorni MG, Casalta JP, Del Zotti F (2015). 2015 ESC Guidelines for the management of infective endocarditis: The Task Force for the Management of Infective Endocarditis of the European Society of Cardiology (ESC). Endorsed by: European Association for Cardio-Thoracic Surgery (EACTS), the European Association of Nuclear Medicine (EANM). Eur Heart J.

[64] Holland TL, Arnold C, Fowler Jr VG. Clinical management of *Staphylococcus aureus* bacteremia: a review. JAMA. 2014;312:1330–41. 10.1001/jama.2014.9743 PMC4263314 25268440

[65] Holubar M, Meng L, Deresinski S. Bacteremia due to methicillin-resistant *Staphylococcus aureus*: new therapeutic approaches. Infect Dis Clin North Am. 2016;30:491–507. 10.1016/j.idc.2016.02.009 27208769

[66] Mendes RE, Tsakris A, Sader HS, Jones RN, Biek D, McGhee P, et al. Characterization of methicillin-resistant *Staphylococcus aureus* displaying increased MICs of ceftaroline. J Antimicrob Chemother. 2012;67:1321–4. 10.1093/jac/dks069 22398650

[67] Imani Fooladi AA, Ashrafi E, Tazandareh SG, Koosha RZ, Rad HS, Amin M (2015). The distribution of pathogenic and toxigenic genes among MRSA and MSSA clinical isolates. Microb Pathog.

[68] Kullar R, Davis SL, Levine DP, Rybak MJ. Impact of vancomycin exposure on outcomes in patients with methicillin-resistant *Staphylococcus aureus* bacteremia: support for consensus guidelines suggested targets. Clin Infect Dis. 2011;52:975–81. 10.1093/cid/cir124 21460309

[69] Jacob JT, DiazGranados CA. High vancomycin minimum inhibitory concentration and clinical outcomes in adults with methicillin-resistant *Staphylococcus aureus* infections: a meta-analysis. Int J Infect Dis. 2013;17:e93–e100. 10.1016/j.ijid.2012.08.005 PMC3780595 23089040

[70] Kalil AC, Van Schooneveld TC, Fey PD, Rupp ME. Association between vancomycin minimum inhibitory concentration and mortality among patients with *Staphylococcus aureus* bloodstream infections: a systematic review and meta-analysis. JAMA. 2014;312:1552–64. 10.1001/jama.2014.6364 25321910

[71] Ludwig F, Edwards B, Lawes T, Gould IM. Effects of storage on vancomycin and daptomycin MIC in susceptible blood isolates of methicillin-resistant *Staphylococcus aureus*. J Clin Microbiol. 2012;50:3383–7. 10.1128/JCM.01158-12 PMC3457431 22855515

[72] Hall 2nd RG, Giuliano CA, Haase KK, Hazlewood KA, Frei CR, Forcade NA, et al. Empiric guideline-recommended weight-based vancomycin dosing and mortality in methicillin-resistant *Staphylococcus aureus* bacteremia: a retrospective cohort study. BMC Infect Dis. 2012;12:104. 10.1186/1471-2334-12-104 PMC3532187 22540223

[73] Hao JJ, Chen H, Zhou JX (2016). Continuous versus intermittent infusion of vancomycin in adult patients: A systematic review and meta-analysis. Int J Antimicrob Agents.

[74] Echeverria-Esnal D, Marin-Casino M, Retamero A, Grau S (2016). Can we guarantee less nephrotoxicity when vancomycin is administered by continuous infusion?. Int J Antimicrob Agents.

[75] Yoon YK, Park DW, Sohn JW, Kim HY, Kim YS, Lee CS, et al. Multicenter prospective observational study of the comparative efficacy and safety of vancomycin versus teicoplanin in patients with health care-associated methicillin-resistant *Staphylococcus aureus* bacteremia. Antimicrob Agents Chemother. 2014;58:317–24. 10.1128/AAC.00520-13 PMC3910721 24165181

[76] Brown AF, Leech JM, Rogers TR, McLoughlin RM. *Staphylococcus aureus* colonization: modulation of host immune response and impact on human vaccine design. Front Immunol. 2014;4:507. 10.3389/fimmu.2013.00507 PMC3884195 24409186

[77] Zasowski EJ, Trinh TD, Claeys KC, Casapao AM, Sabagha N, Lagnf AM, et al. Multicenter observational study of ceftaroline fosamil for methicillin-resistant *Staphylococcus aureus* bloodstream infections. Antimicrob Agents Chemother. 2017;61(2). doi:10.1128/AAC.02015-16. 10.1128/AAC.02015-16 PMC5278749 27895012

[78] Moise PA, Forrest A, Birmingham MC, Schentag JJ. The efficacy and safety of linezolid as treatment for *Staphylococcus aureus* infections in compassionate use patients who are intolerant of, or who have failed to respond to, vancomycin. J Antimicrob Chemother. 2002;50:1017–26. 10.1093/jac/dkf215 12461026

[79] Park HJ, Kim SH, Kim MJ, Lee YM, Park SY, Moon SM, et al. Efficacy of linezolid-based salvage therapy compared with glycopeptide-based therapy in patients with persistent methicillin-resistant *Staphylococcus aureus* bacteremia. J Infect. 2012;65:505–12. 10.1016/j.jinf.2012.08.007 22902942

[80] Sander A, Beiderlinden M, Schmid EN, Peters J (2002). Clinical experience with quinupristin-dalfopristin as rescue treatment of critically ill patients infected with methicillin-resistant staphylococci. Intensive Care Med.

[81] Wilson SE, Graham DR, Wang W, Bruss JB, Castaneda-Ruiz B. Telavancin in the treatment of concurrent Staphylococcus aureus bacteremia: a retrospective analysis of ATLAS and ATTAIN studies. Infect Dis Ther. 2017 Jul 10. doi:10.1007/s40121-017-0162-1. [Epub ahead of print]. 10.1007/s40121-017-0162-1 PMC5595776 28695347

[82] Paul M, Bishara J, Yahav D, Goldberg E, Neuberger A, Ghanem-Zoubi N, et al. Trimethoprim-sulfamethoxazole versus vancomycin for severe infections caused by meticillin resistant *Staphylococcus aureus*: randomised controlled trial. BMJ. 2015;350:h2219. 10.1136/bmj.h2219 PMC4431679 25977146

[83] Davis JS, Sud A, O'Sullivan MVN, Robinson JO, Ferguson PE, Foo H, et al. Combination of vancomycin and beta-lactam therapy for methicillin-resistant *Staphylococcus aureus* bacteremia: a pilot multicenter randomized controlled trial. Clin Infect Dis. 2016;62:173–80. 10.1093/cid/civ808 26349552

[84] Shafiq I, Bulman ZP, Spitznogle SL, Osorio JE, Reilly IS, Lesse AJ, et al. A combination of ceftaroline and daptomycin has synergistic and bactericidal activity in vitro against daptomycin nonsusceptible methicillin-resistant *Staphylococcus aureus *(MRSA). Infect Dis (Lond). 2017;49:410–6. 10.1080/23744235.2016.1277587 28116950

[85] Calfee DP, Salgado CD, Milstone AM, Harris AD, Kuhar DT, Moody J, et al. Strategies to prevent methicillin-resistant *Staphylococcus aureus* transmission and infection in acute care hospitals: 2014 update. Infect Control Hospital Epidemiol. 2014;35:772–96. 10.1086/676534 24915205

[86] Marimuthu K, Pittet D, Harbarth S (2014). The effect of improved hand hygiene on nosocomial MRSA control. Antimicrob Resist Infect Control.

[87] Jinadatha C, Quezada R, Huber TW, Williams JB, Zeber JE, Copeland LA. Evaluation of a pulsed-xenon ultraviolet room disinfection device for impact on contamination levels of methicillin-resistant *Staphylococcus aureus*. BMC Infect Dis. 2014;14:187. 10.1186/1471-2334-14-187 PMC3986444 24708734

[88] Salgado CD, Sepkowitz KA, John JF, Cantey JR, Attaway HH, Freeman KD (2013). Copper surfaces reduce the rate of healthcare-acquired infections in the intensive care unit. Infect Control Hosp Epidemiol.

[89] Kavanagh KT, Calderon LE, Saman DM, Abusalem SK. The use of surveillance and preventative measures for methicillin-resistant *Staphylococcus aureus* infections in surgical patients. Antimicrob Resist Infect Control. 2014;3:18. 10.1186/2047-2994-3-18 PMC4028005 24847437

